# Very low error rates of NGS-based HLA typing at stem cell donor recruitment question the need for a standard confirmatory typing step before donor work-up

**DOI:** 10.1038/s41409-018-0411-2

**Published:** 2018-11-30

**Authors:** Daniel M. Baier, Jan A. Hofmann, Heike Fischer, Gabi Rall, Julia Stolze, Katja Ruhner, Vinzenz Lange, Jürgen Sauter, Alexander H. Schmidt

**Affiliations:** 1 DKMS, Kressbach 1, Tübingen, Germany; 2 DKMS Life Science Lab, Blasewitzer Straße 43, Dresden, Germany

**Keywords:** Genomics, Haematopoietic stem cells, Genetics

Allogeneic hematopoietic stem cell transplantation (HSCT) is a curative therapy for severe blood diseases. According to the EBMT activity survey, unrelated stem cell donors provided 52% of all allogeneic stem cell products in 2015 [1]. Unrelated donor registries inform, recruit and administer potential donors and have built up a global pool of currently more than 32 million donors since the 1970s [2].

As an essential part of the donor recruitment process, the potential donor’s HLA genotype is determined. The specification of typing scope (number of loci, resolution) and typing method (e.g., Sanger sequencing, next-generation sequencing (NGS)) is made by donor registries under consideration of limited resources for donor registry operations. NGS technologies offer high-throughput high-resolution HLA typing of new donors at unprecedented low costs [3–6].

The process leading to stem cell donation currently includes a mandatory confirmatory typing (CT) (sometimes also named verification typing (VT)) step. Main purpose of the CT step is the confirmation of the potential donor’s HLA typing result by typing a new donor sample, generally at the same lab that executed the prospective transplant recipient’s HLA typing. In many, albeit not all, cases of discrepancies between the original and confirmatory typing results, the donor registry administering the respective donor and providing the CT sample is informed accordingly. At DKMS, we resolve cases with at least one discrepant HLA locus, e.g., by collecting a third donor sample and mandating a “neutral” HLA lab (different from the labs carrying out the original typing and the CT) to type the sample. Based on the result, either the original typing result or the CT result is classified as erroneous. Discrepancies without relevance for donor-patient HLA matching (e.g., silent mutations outside exons coding for the antigen recognition domain) sometimes remain unresolved. For the analyses in this letter, only samples and loci were considered for which recruitment typing and CT typing results were available.

Apart from confirmation or falsification of the donor’s original HLA typing result, the CT step also comprises a phone contact with the potential donor including the provision of detailed information on the two stem cell donation methods, the clarification of the donor’s continued interest to donate, the analysis of infectious disease markers (IDM), and a questionnaire-based health screening. In urgent cases, the CT step can be merged with the following step in the process leading to stem cell donation, the so-called donor work-up (WU). Donor WU includes, for example, the donor’s final medical clearing at the collection center. In 2016, DKMS Germany received 27,232 CT requests, 6825 work-up requests and 412 combined requests (named “CT/work-up parallel requests” (CWP requests) in the following). Of the CT requests, 4924 (18.1%) finally led to a donation while further 116 requests (0.4%) were still open at the time of data retrieval. Furthermore, 227 CWP requests (55.1%) resulted in a donation with no requests open anymore.

In this letter, we present real-life data from the DKMS Germany donor file regarding discrepancies between HLA typing results at donor recruitment and CT results. Primarily, we focus on donors who were typed by NGS-based methods at the DKMS Life Science Lab (Dresden, Germany) where NGS had been established as high-throughput method for donor typing at recruitment in 2013 [3, 6]. Until 31 March 2017, 2,055,028 newly registered DKMS German donors were typed by NGS for 6 HLA loci (HLA-A, -B, -C, -DRB1, -DQB1 and -DPB1).

From 1 July 2013 to 31 May 2017, there were 39,591 CT requests for donors of that group. In 30,947 of these cases, a CT sample was provided, yielding 140,636 HLA typing results for validation (25,990 typing results for HLA-A, 25,979 for HLA-B, 25,913 for HLA-C, 26,022 for HLA-DRB1, 25,494 for HLA-DQB1, and 11,238 typing results for HLA-DPB1). Differences in the numbers of typing results for validation originated from different typing profiles at CT or incomplete reporting of CT results to the donor registry. Especially the HLA-DPB1 locus was often not included in the CT profile.

In order to compare various typing methods, in addition we analyzed all incoming 30,944 CT requests (23,190 CT samples with 83,551 typing results) of the same period (1 July 2013 to 31 May 2017). Allocation of original HLA typing data to the various typing methods was based on available donor registry information including date of typing and nature of HLA typing data. For 27,447 typing results provided, the allocation to a typing method was not definitely possible. These cases were excluded from further analysis. For 56,104 analyzed typing results, 9700 were originally processed by SSO and 46,404 by Sanger sequencing.

**Fig. 1:**
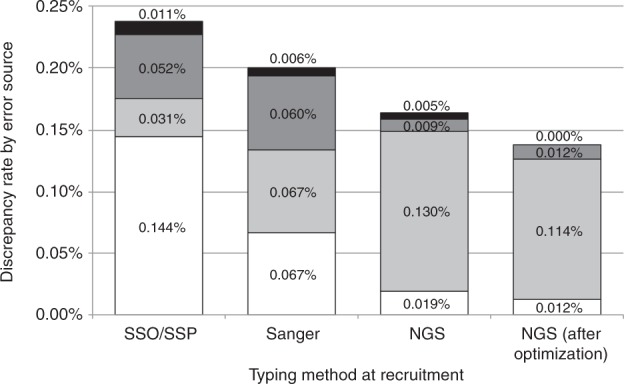
Discrepancy rates by typing method at donor recruitment and by error source. White: typing at recruitment erroneous. Light gray: CT erroneous. Dark gray: sample switch. Black: error source unresolved

For each of the three genotyping technologies we calculated the rate of discrepancies based on the CT results and the respective error source (Fig. 1). When the typing at recruitment had been carried out using NGS, the total locus-wise discrepancy rate was 0.163% (230 of 140,709 typing results, column #3). In 79.6% of the discrepant cases the CT result turned out to be wrong (error rate: 0.130%). In 27 cases (11.7%) the error was due to NGS typing at recruitment, yielding a nearly 7-fold lower error rate of 0.019%. In 13 cases (5.7%) the discrepancy occurred due to a sample switch (error rate: 0.009%) while in 7 cases (3.0%) the discrepancy could not be resolved. Error rates for Sanger sequencing and SSO/SSP typing at recruitment were about 3.5-fold and 7.5-fold higher than for NGS. Interestingly, CT error rates strongly depended on the typing method at recruitment. While strange at first sight, this observation can be easily explained by increasing typing resolution from SSO/SSP typing via Sanger sequencing to NGS that makes it more difficult to obtain a CT result that is consistent with the original typing. Similar error rates for typing at recruitment and CT in the Sanger column suggest most CTs were carried out using Sanger sequencing in the study period.

Of the 27 erroneous NGS typing results, 16 concerned the HLA-DPB1 locus, 5 HLA-DQB1, 3 HLA-A, 1 HLA-B, 1 HLA-C, and 1 HLA-DRB1. Issues with homozygosity handling were responsible for 14 of the 16 HLA-DPB1 typing errors. We observed this specific problem in a preliminary analysis in 2015 and accordingly improved our neXtype software [3, 6] by adjusting the detection parameters for hetero- and homozygosity. Since then, no additional errors due to false detection of homozygosity in HLA-DPB1 have been observed. Column #4 shows NGS error rates from donors who were initially typed after this optimization step (955,362 of 2,055,028 donors; beginning 14 September 2015). Within this sub-group, an error in NGS typing at recruitment occurred in 4 of 32,536 typing results (error rate: 0.012%).

Taken together, NGS typing data showed very low error rates in our practical retrospective study. These results underline the appropriateness of NGS for upfront HLA typing in the donor registry setting. Apart from the low error rates being in the focus of this work, further advantages of the NGS approach compared to Sanger sequencing include lower costs and less data ambiguities, the possibility to extend the typing profile in an easy and cost-effective way [7, 8], and easier detection and determination of new alleles [9]. These facts make a strong case for NGS-based upfront HLA typing of newly registered stem cell donors.

In most cases of a discrepancy between an NGS-based original typing result and a CT result, the CT result turned out to be wrong while the original NGS result was indeed correct. According to our analyses, about 3500 typing results have to be processed at CT to identify one erroneous upfront HLA typing result or sample switching. Among these 3500 typing results processed, about 4.5 are expected to be typed incorrectly at CT according to our data. Therefore, the current standard CT step before donor work-up seems questionable for donors with NGS-based HLA typing at recruitment as it increases the duration of the donor search process, requires an additional blood drawing from the donor and is associated with significant costs, e.g., for – often intercontinental – shipping of the CT sample. At first sight, establishing CWP as standard process for donors with NGS-based original typing seems to be an appropriate solution. However, also in donor registries with high donor availability about 20% of the requested donors turn out to be not available during the CT step. It seems problematic to shift the identification of these donors into the donor work-up step. As the original purpose of CT, the identification of erroneous upfront HLA typing results, is, however, of negligible relevance for NGS-typed donors, we propose to establish a new pre-WU process step (Health and availability check (HAC)) that includes a phone contact with the potential donor, the provision of detailed information on the donation process, clarification of the donor’s continued interest and availability to donate, a questionnaire-based health screening but not the collection and shipping of a CT sample. Instead, re-typing of all potential donors with prior NGS typing would become part of the WU step.

The introduction of the new HAC process step by donor centers and registries after clearance with transplant physicians and search coordinators should decrease duration and costs of the donor search process as well as inconvenience for potential donors. These improvements would be substantial as currently only about 25% of all donors with a CT request proceed to donor WU. As a disadvantage of the HAC establishment, some complexity would be added to the search process as the current CT step would not be fully replaced by HAC but remain as an option, e.g., for donors not typed by NGS. Therefore, it would become necessary to provide some information on the typing technology applied for each donor (at least NGS vs. non-NGS). It could also be defined that the new HAC process step is open only for NGS-typed donors from registries with error rates (including sample switching) for such donor typing below a certain threshold. Of course, the use of the new HAC process step would be at the discretion of transplant physicians and search coordinators. For example, even potential donors with prior NGS typing are normally not typed for HLA-DQA1, -DPA1, or -DRB3/4/5. Besides, their typing results generally do not exclude all known null alleles. Transplant physicians who wish to include these parameters in their selection decision may prefer the common CT step to collect these data before they request a donor for WU. As our donor registry practice shows that the analysis of IDM results only rarely prevents prospective donors from donating stem cells, this part of the current CT step could also be shifted to the WU step, thus requiring no blood withdrawal at all during the proposed new pre-WU process step.
